# 

**Published:** 1932

**Authors:** 


### CONTENTS.

| | Page |
|---|---|
| Some Observations on the Relationship between Structure and Function. By J. R. Charles, M.A., M.D., F.R.C.P. | 1 |
| Human Improvability. By Charles S. Myers, C.B.E., F.R.S., M.A., M.D., D.Sc. | 31 |
| Trigeminal Neuralgia. By H. H. Carleton, M.D., M.R.C.P. | 47 |
| Some Observations on the Relative Efficacy of the Various Treatments of Pernicious Anæmia. By G. L. Ormerod, M.A., M.B., B.Chir. | 61 |
| Reviews of Books | 87 |
| Editorial Notes | 95 |
| Meetings of Societies | 99 |
| The Medical Library of the University of Bristol | 103 |
| Local Medical Notes | 105 |

